# Systematic Evaluation of Different Coating Chemistries Used in Thin-Film Microextraction

**DOI:** 10.3390/molecules25153448

**Published:** 2020-07-29

**Authors:** Jia-Wei Liu, Khaled Murtada, Nathaly Reyes-Garcés, Janusz Pawliszyn

**Affiliations:** Department of Chemistry, University of Waterloo, 200 University Avenue West, Waterloo, ON N2L 3G1, Canada; liujiawei19871995@163.com (J.-W.L.); kmurtada@uwaterloo.ca (K.M.); nathalyreyesg@gmail.com (N.R.-G.)

**Keywords:** thin-film microextraction, LC-MS/MS, carbon-based sorbents, SPE-based sorbents

## Abstract

A systematic evaluation of eight different coatings made of solid phase extraction (SPE) and carbon-based sorbents immobilized with polyacrylonitrile in the thin-film microextraction (TFME) format using LC-MS/MS was described. The investigated coatings included graphene, graphene oxide, multi-walled carbon nanotubes (MWCNTs), carboxylated MWCNTs, as carbon-based coatings, and polystyrene-divinylbenzene (PS-DVB), octadecyl-silica particles (C18), hydrophilic–hydrophobic balance particles (HLB) and phenyl-boronic acid modified particles (PBA), as SPE-based coatings. A total of 24 compounds of diverse moieties and of a wide range of polarities (log P from −2.99 to 6.98) were selected as probes. The investigated coatings were characterized based on their extraction performance toward the selected probes at different pH values and at optimized desorption conditions. In the case of SPE-based coatings, PS-DVB and HLB exhibited a balanced extraction for compounds within a wide range of polarities, and C18 showed superior extraction recoveries for non-polar analytes. Carbon-based coatings showed high affinity for non-polar compounds given that their main driving force for extraction is hydrophobic interactions. Interestingly, among the studied carbon-based coatings, graphene oxide showed the best extraction capabilities toward polar compounds owing to its oxygen-containing groups. Overall, this work provided important insights about the extraction mechanisms and properties of the investigated coatings, facilitating the coating selection when developing new TFME applications.

## 1. Introduction

Thin-film microextraction (TFME), an alternative configuration of solid-phase microextraction (SPME), was first introduced in 2003 [1] and has since been employed for various LC and GC applications [1,2,3,4]. The merit of the TFME format is the increase in the volume of extracting phase, which in some cases may improve the analyte recoveries and enhance the sensitivity for trace level analysis [5,6]. In LC applications, the extracting phase is immobilized on a blade-shaped substrate instead of the traditional rod-shaped geometry, providing a higher surface area-to-volume ratio which enables an increase in the extracting phase volume, and consequently allows for improved extraction efficiency [7]. Moreover, multiple thin films can be easily assembled in a 96-well plate compatible format to achieve high-throughput sample preparation [8,9,10].

Careful selection and evaluation of extracting phases play an important role in TFME method development. Unfortunately, the limited information about the relations of various extracting phases with different compounds is still challenging in the TFME technique. For instance, commercially available adsorbents typically used in solid phase extraction (SPE) can be employed as coating materials, such as C18 particles [8], polystyrene-divinylbenzene (PS-DVB) [11], and phenylboronic acid (PBA) [12], among others. Mousavi et al. systematically evaluated these SPE sorbents for high-throughput analysis of Escherichia coli metabolomics in the 96-blade format. However, the effect of extraction conditions, e.g., pH, on the TFME extraction was not performed in this work [13]. In addition to the above SPE phases, carbon nanomaterials including graphene and carbon nanotubes (CNTs) have been employed as potential adsorbents for SPE and SPME applications due to their large surface area and tunable surface properties [14,15,16,17]. In this regard, CNTs have been employed in TFME coatings for high-throughput analysis of phenolic compounds in water [18]. However, evaluations of the extraction abilities of carbon nanomaterials as thin-film coatings for LC-MS analysis of various types of compounds are still limited. In addition, the effect of extraction conditions, such as pH, on the extraction performance of TFME coatings must be thoroughly evaluated to provide analysts with a better understanding of the extraction behaviors of various kinds of compounds on these adsorbents. Such information would help better inform analysts with respect to coating selection during TFME method development of real applications.

In this study, eight different types of adsorbents were evaluated as TFME coatings for LC-MS analysis. These adsorbents can be divided into two classes, namely the carbon-based coatings graphene, graphene oxide, multi-walled carbon nanotubes (MWCNTs) and carboxylated MWCNTs (MWCNTs-COOH), and the SPE-based coatings PS-DVB, C18, PBA and hydrophilic–lipophilic balance (HLB). Generally, various kinds of interactions between adsorbents and compounds, such as hydrophobic, hydrophilic, electrostatic, π-π stacking, and covalent bonding, are all involved in these materials, indicating their representativeness among plenty of materials. These adsorbents were further coated onto 96-blades through a developed spraying method with polyacrylonitrile (PAN) as glue [8]. A total of 24 compounds from different chemical classes and of widely varying polarities (log P of −2.99 to 6.98) were selected as probes for the coating evaluation. Extractions were performed at physiological pH conditions and the desorption solvent was optimized to achieve favorable extraction efficiency. The influence of pH (3.0, 7.4 and 10.0) on the extraction capacities of the selected coatings was further investigated in order to better elucidate the extraction mechanisms of these analytes on the eight different coatings.

## 2. Results and Discussion

### 2.1. Optimization of Desorption Solvent for Coating Evaluation

**Figure 1:**
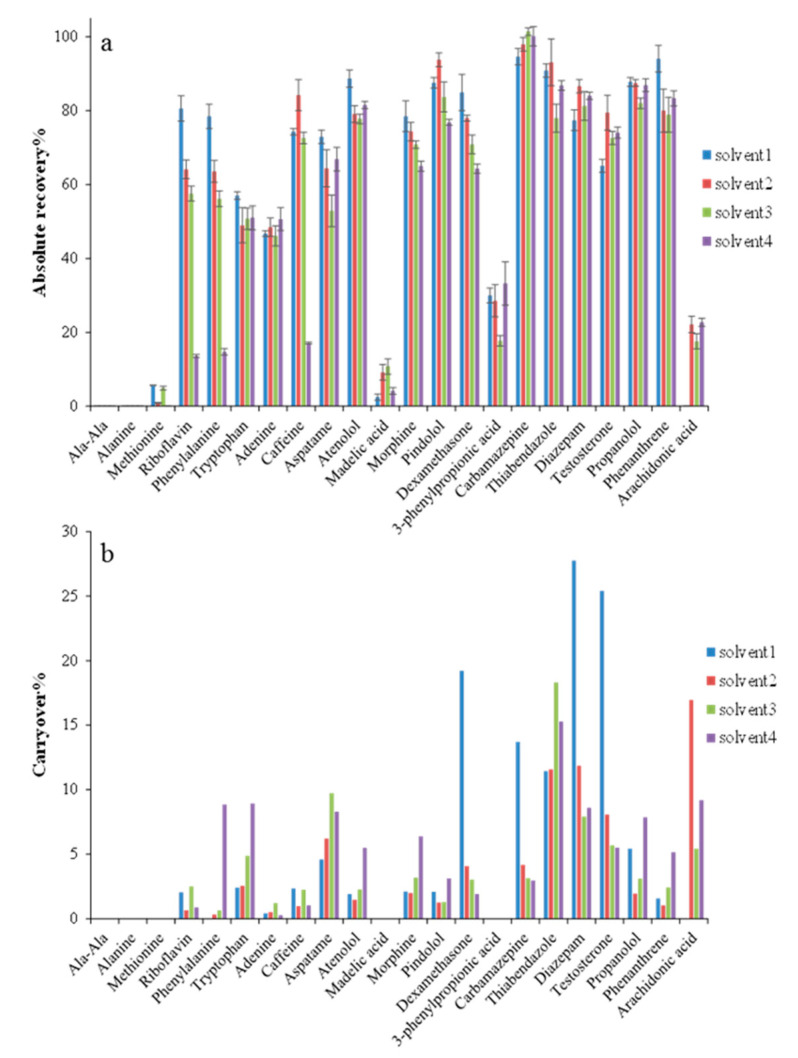
Absolute recovery (**a**) and carryover values (**b**) obtained for each model analyte when using a polystyrene-divinylbenzene (PS-DVB) coating and four different desorption solvents. Solvent 1: methanol/acetonitrile/water (25/25/50, *v*/*v*/*v*), solvent 2: methanol/acetonitrile/water (40/40/25, *v*/*v*), solvent 3: methanol/acetonitrile (80/20, *v*/*v*), solvent 4: acetonitrile/water (80/20, *v*/*v*). Formic acid (0.1%, *v*/*v*) was added to all the desorption solvents. Extractions were performed in triplicate at pH 7.4 for 2 h, and desorption time was 1 h.

The first part of this study comprised the optimization of desorption conditions for all the investigated extraction phases. Minimum carryover and complete desorption of the testing probes was essential to ensure reliable data to characterize the studied coatings. In this case, four different desorption solvents including methanol/acetonitrile/water (25/25/50, *v*/*v*/*v*, solvent 1), methanol/acetonitrile/water (40/40/20, *v*/*v*/*v*, solvent 2), methanol/acetonitrile (80/20, *v*/*v*, solvent 3), and acetonitrile/water (80/20, *v*/*v*, solvent 4) acidified with formic acid (0.1%, *v*/*v*) were evaluated. Figure 1 shows results corresponding to absolute recoveries and carryover values found for the PS-DVB coating (data corresponding to other coating chemistries is listed in Table S1). As can be seen in Figure 1, among the tested solvents, methanol/acetonitrile/water (40/40/20, *v*/*v*/*v* solvent 2) showed the best desorption efficiency, the lowest carryover values for the majority of the tested compounds, and a good compromise in terms of chromatographic separation. Therefore, methanol/acetonitrile/water (40/40/20, *v*/*v*/*v*) with formic acid (0.1%, *v*/*v*) was employed as desorption solvent for the following coating evaluation experiments.

### 2.2. Comparison of Extraction Efficiency of Various Coatings at pH 7.4

**Figure 2:**
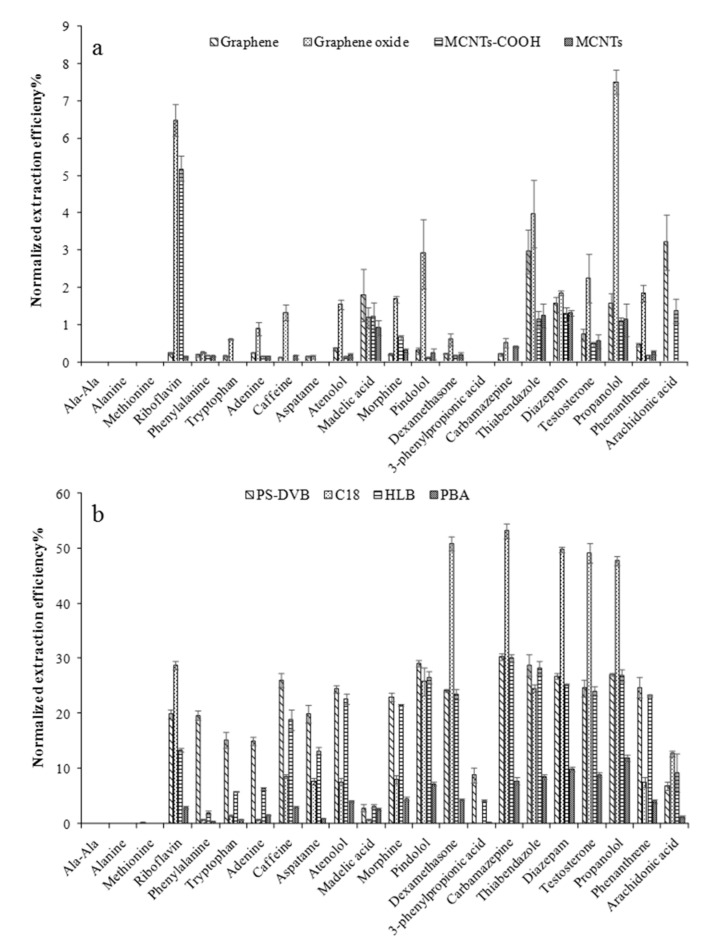
Comparison of extraction performances of the eight coatings for all analytes at pH 7.4, (**a**) carbon-based coatings, (**b**) solid phase extraction (SPE)-based coatings. Extractions were performed in triplicate for 2 h, and desorption was performed for 1 h using desorption solvent 2.

Figure 2 presents normalized extraction efficiencies obtained for each analyte with all the studied coatings (the normalization method of extraction efficiencies was seen in Table S2). Overall, SPE-based coatings showed better extraction performance toward most of the selected probes in comparison to carbon-based coatings. No extraction or negligible recovery of Ala-Ala (<0.1%), alanine (<0.1%) and methionine (<0.5%) at physiological pH in all cases evidenced the poor extraction capabilities of the studied coatings toward highly polar small compounds. Among all the extraction phases, the lowest recoveries for most polar probes were observed for carbon-based coatings (Figure 2a). These carbon-based sorbents are characterized for their highly hydrophobic surfaces, and therefore improved extraction efficiencies along with increasing log P values are expected. Additionally, the delocalized π-electron system of such materials allows for π-π interactions, leading to high affinity toward compounds with aromatic rings [16]. Considering these features, effective extraction of compounds such as diazepam, propranolol, testosterone, phenanthrene, and arachidonic acid was expected. However, all coatings prepared with carbon-based sorbents showed normalized extraction recoveries below 7%, and particularly graphene and MWCNTs displayed recoveries under 3% (Figure 2a). These substantially low values might be possibly attributed to agglomeration or aggregation of graphene sheets or MWCNTs during the coating preparation, which deteriorates their sorptive characteristics and effective elution of extracted analytes, as reported elsewhere [16,19]. As a practical solution to this issue, covalent binding of graphene and graphene oxide to silica particles was proposed [20]. Although, in this study, graphene-based materials were immobilized in an open-bed geometry using PAN as glue, it would be valuable to further determine the possible effect of aggregation by comparing the performance of the already evaluated graphene and graphene oxide coatings with others prepared using silica-bound graphene.

As shown in Figure 2a (dot bar), the graphene oxide coating showed the best performance among all tested carbon-based extraction phases. As a matter of fact, graphene oxide and MWCNTs-COOH own in their structure a high density of oxygen-containing functional groups. These groups add hydrophilicity to such materials, hence favoring the extraction of more polar compounds due to electrostatic and hydrogen-bond interactions [17]. Normalized recoveries observed for riboflavin, for instance, evidence the affinity of graphene oxide and MWCNTs-COOH coatings toward multiple hydroxyl moieties via hydrogen-bond interactions. It is also worth emphasizing that besides improving the affinity of the coating for more polar functionalities, the oxygenated groups on the graphene oxide surface also facilitate their desorption process [16]. Polar moieties on the structure of such sorbents reduce hydrophobic interactions, which can still be considered as the main contributors to the extraction process.

In the case of SPE-based extraction phases, PS-DVB and HLB coatings yielded balanced coverage for hydrophilic and hydrophobic compounds, and similar normalized extraction recoveries for all the tested probes. The main noticeable difference between HLB and PS-DVB was observed for the extraction of phenylalanine, tryptophan, and adenine, where PS-DVB showed superior performance compared to HLB. These three compounds are characterized as small polar molecules (molecular weight below 205 Dalton) bearing in their structure aromatic moieties and amine groups. Although both PS-DVB and HLB sorbents contain divinylbenzene functionalities able to display the π-π type of interactions, the smaller pores of PS-DVB (50 Å for PS-DVB and 80 Å for HLB particles) may have probably allowed for improved extraction of the aforementioned compounds. Similar results corresponding to PS-DVB performance have already been reported by Vuckovic et al. [21].

The C18 extraction phase, in turn, showed poor extraction capabilities toward the most polar probes (log P values under 1) and, as expected, superior normalized recoveries for compounds of medium to high hydrophobicity, namely dexamethasone, carbamazepine, diazepam, testosterone, and propranolol. As shown in Figure 2b (dot bar), hydrophobic interactions provided by C18 particles were sufficient to allow for an almost two-fold increase in normalized recoveries as compared to HLB and PS-DVB coatings. Another factor that should also be considered regards the differences in particle size. The small size of C18 particles (5 μm) permits for more efficient arrangement of the sorbent in a given volume, and this, in turn, might result in higher normalized recoveries. The only one exception to this phenomenon was phenanthrene; although characterized by a high log P value (4.46) it yielded a lower recovery by the C18 coating in comparison to the results obtained using PS-DVB and HLB phases. The aromatic-ring-rich structure of phenanthrene provides stronger π-π interactions with PS-DVB or HLB phases rather than the C18 phase. In addition, it is worth mentioning that riboflavin showed a normalized recovery different from the expected trend. As presented in Figure 2, among all the tested extraction phases, the highest normalized recovery for riboflavin was obtained with C18. Taking into account the high polarity of this compound (log P −1.46), it is reasonable to consider that the ion-exchange capacity provided by the low concentration of silanol groups present in the silica may play an important role during the extraction process of basic solutes (e.g., riboflavin) [22].

For the PBA coating, the multiple functional groups provide different types of interactions including hydrogen bonding interactions, ionic interactions, as well as Van der Waals and π-π interactions for various kinds of substances [12]. Therefore, the PBA coating offers universal extraction capacities, toward both polar and non-polar compounds. However, the much lower normalized extraction efficiencies of the PBA coating are attributed to its having the largest coating volume among these four SPE-based sorbents, resulting in the lowest correction factor for calculation. Moreover, in comparison to PS-DVB (owning same particle size with PBA), the density of phenyl-boronic acid groups grafted on PBA-modified silica particles would provide less abundant adsorptive sites, facilitating much lower extraction efficiency.

### 2.3. pH Effect on the Extraction Performance of Different Coatings

Table S3 illustrates results corresponding to the extraction performance of the studied coatings at different pH values (3.0, 7.4 and 10.0). As can be seen, different trends for each coating and for each analyte were found at such conditions. First of all, highly polar compounds bearing on their structure carboxylic and amine moieties, e.g., alanine and Ala-Ala, could be extracted by all the coatings under both acidic and basic conditions. For carbon-based coatings, under acidic condition (pH 3.0), protonated amino groups (-NH_3_^+^) could provide weak cation-π [23] types of interactions with conjugated carbon rings of adsorbents, which might contribute to the adsorption process. For basic conditions (pH 10.0), it is postulated that deprotonated carboxylate groups would strengthen the dipole–dipole interactions between amino acids and conjugated carbon-substrates in comparison to their nonionic counterparts at pH 7.4 [24], thus facilitating the extraction of amino acids onto these adsorbents. Similar results were also observed for extraction of phenylalanine and tryptophan by using carbon-based coatings. For SPE-based coatings, the increase in extraction efficiencies towards polar amino acids under acidic and basic conditions indicated that there were weak ionic interactions between amino acids and the sorbents. It should be considered that the presence of polar moieties in the structures of all SPE sorbents, i.e., C-N and C=O for HLB, N-H and B-OH for PBA, residual Si-OH for C18 and functional polar groups for PS-DVB, might provide hydrophilic or weak ion exchange interactions with ionogenic amino acids in acidic or basic solutions, thus improving the extraction of amino acids onto SPE-based coatings. Another interesting observation was the dramatic increase in amounts of mandelic acid extracted by all coatings when pH was adjusted to 10. Mandelic acid contains a hydroxyl group that is very close to its carboxylic group, thus strong intra-molecular hydrogen bonds are formed between hydroxyl and carboxyl groups [25]. As the pH of the matrix increases, the intra-molecular hydrogen bond force would weaken and completely disappear under strong basic conditions [26], which might improve the hydrogen bonding and π-π electron donor-acceptor type of interactions between mandelic acid and adsorbents, and thus give rise to great improvements in extraction. Thirdly, the significant decrease in extraction efficiencies for riboflavin and aspartame by all coatings at pH 10.0 could be attributed to their instability and rapid degradation [27,28] under strong basic conditions.

Apart from the results discussed in the above section, several differences in extraction performance were observed for several compounds when using carbon-based coatings and SPE-based coatings under various pH conditions. These variations serve as an indication of the different interactions taking place between the studied analytes and the surface of each coating. In carbon-based coatings, graphene, MWCNTs, and MWCNTs-COOH exhibited a similar extraction trend for all model compounds at the studied pHs. This observation supports that these analytes were adsorbed onto the surface of these three coatings through similar binding forces, mainly hydrophobic and π-π types of interactions. Notably, arachidonic acid showed great enhancement of extraction on these coatings at pH 3.0. This indicates that protonated arachidonic acid preferred to be adsorbed onto the hydrophobic surfaces of carbon-based coatings. Improvements in the extraction of some drugs, i.e., atenolol, pindolol, and propranolol, were also observed for carbon-based coatings at pH 10.0. It should be considered that the adsorption/extraction of these drugs on hydrophobic surfaces is based on two factors, i.e., the hydrophobicity of the drug (conditioned by the value of log P) and the ionization of the molecule (determined by the values of pH and pKa). Therefore, log D is a pH-dependent modified log P value and is relevant for compounds that are partly dissociated or protonated [29]. It can be calculated as Equations (1) and (2) [30]:

For acidic molecules, log D is determined as:

log D = log P − log(1 + 10^(pH-pKa)^), (1)

Whereas for basic molecules,

log D = log P − log(1 + 10^(pKa-pH)^), (2)

For example, the modified log D is:

The D values of propranolol at pH 3.0, 7.4, and 10.0 were found to be −2.94, 1.47, and 3.38, respectively. As pH value increases, the hydrophobicity of propranolol gradually increases, thus facilitating higher extraction efficiency through hydrophobic interactions with these coatings. Moreover, atenolol and pindolol showed the same variation tendencies of log D values with increasing pH values, and improvements in extraction were also obtained. Similar trends for these three drugs adsorbed on the C18 coating at different pHs were also observed due to the hydrophobicity of the C18 phase. For the graphene oxide coating, the best extraction performances for most analytes took place under acidic and basic conditions, demonstrating that alternative binding forces i.e., hydrogen bonds under acidic conditions [31] and electrostatic interactions under basic conditions [32], respectively, contributed to extraction.

For SPE-based coatings, PS-DVB and HLB coatings showed a similar tendency toward most of the analytes with respect to variations in pH values. As the C18 coating is strongly hydrophobic, no significant changes were observed under various pH conditions in relation to trends of analytes with high log P values (dexamethasone, carbamazepine, thiabendazole, diazepam and testosterone) adsorbing on the C18 coating. For the PBA coating, the most favorable recoveries occurred at physiological conditions due to the multiple interactions between analytes and PBA particles. It should be mentioned that the dissociation of boronate moieties occurs under basic conditions (pKa 8.8); thus, the negatively charged surface of PBA at pH 10 was favorable for extraction of compounds containing N-H groups such as atenolol and pindolol. However, compounds with hydroxyl groups or negative charges were less extracted by the PBA coating (phenylalanine, tryptophan, morphine and dexamethasone). The extraction efficiency of 3-phenylpropionic acid was observed to significantly increase at pH 3.0 for all SPE-based coatings. It is reasonable that 3-phenylpropionic acid is protonated under strong acidic conditions, making it easier to be extracted onto SPE-based coatings in its protonated form. In contrast, for arachidonic acid (log P 6.98), recoveries were lower under acidic conditions in comparison to recoveries under neutral and basic conditions when SPE-based coatings were used. These results would indicate that depronated arachidonic acid shows higher affinity toward both PS-DVB and HLB coatings due to the weak ion-exchange properties of PS-DVB and the hydrophilic moieties of HLB. Besides, the silanol groups present in the silica support of the C18 and PBA adsorbents were effectively activated under strong basic conditions, thus providing alternative hydrophilic interactions with deprotonated arachidonic acid molecules, and consequently resulting in higher extraction efficiencies.

## 3. Materials and Methods

### 3.1. Chemicals, Materials and Solutions

Detailed information regarding chemicals and materials used in this work is available in the Supplementary Materials (Section S1.1). A summary of the probe compounds, with their physicochemical characteristics and the quantitation ions, is shown in Supplementary Materials Table S4. Details related to the preparation of stock and working solutions are available in the Supplementary Materials (Section S1.2).

### 3.2. Preparation of TFME Coatings

**Figure 3:**
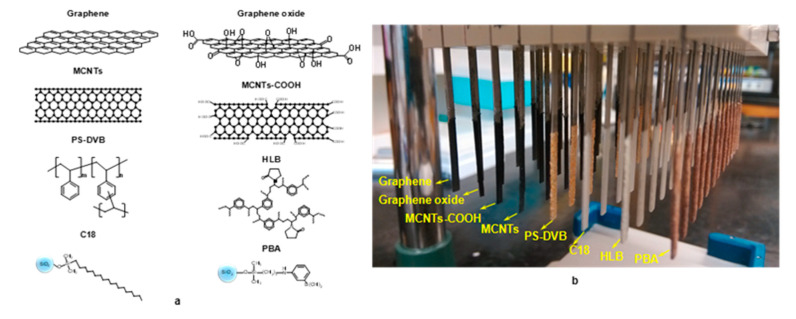
(**a**) Structural scheme of eight different types of adsorbents and (**b**) photograph of thin-film coatings prepared from eight different adsorbents.

TFME blades were prepared by following the coating procedure already described elsewhere [8]. Briefly, stainless steel blades were etched with hydrochloric acid and thoroughly washed with nanopure water. Afterwards, TFME coatings were applied on the stainless steel by spraying uniform layers of particles/PAN/DMF slurry. Curing at high temperature (150–180) was carried out after applying each layer. Multiple spraying–curing cycles were performed until the desired coating thickness was attained. Details regarding the particles/PAN ratios used in preparation of the coatings are listed in Table S5. In the case of graphene oxide, particles were first dispersed in DMF using a XL-2000 series Misonix sonicator (Qsonica LLC, Newtown, CT, USA) before adding the PAN glue solution. Figure 3 shows the structures of the different extraction phases used, and presents the coatings prepared according to the procedure already described.

### 3.3. TFME Procedure for Coating Evaluation

The evaluation was performed with the use of a manual Concept 96 kit (Professional Analytical System (PAS) Technology, Magdala, Germany) [18]. The coating evaluation procedure was carried out in four steps. First, all coatings were pre-conditioned in methanol/water (50/50, *v*/*v*) solution for 30 min. Next, 1.5 mL of a working solution (spiked buffers) was transferred into a 2.0 mL 96-well plate (VWR International, Mississauga, ON, Canada), and extractions were conducted under 850 rpm for 2 h. Following this, the extracted analytes were desorbed by immersing the coated blades in 1.5 mL of desorption solvent under constant agitation conditions (850 rpm) for 1 h. In order to investigate the carryover of each coating as well as achieve complete desorption of analytes from coatings, a second desorption step was conducted in 1.5 mL of fresh desorption solvent. All desorption solutions were stored at 4 °C and injected into the LC-MS/MS system for instrumental analysis.

### 3.4. Liquid Chromatography and Tandem Mass Spectrometry Conditions

Details on analytical instrumentation are shown in the Supplementary Materials (Section S1.3). Optimized tuning parameters for each compound and LC-MS/MS method conditions for positive and negative ionization modes, respectively, are summarized in Tables S6 and S7.

## 4. Conclusions

In this study, eight different adsorbents used as TFME coatings were systematically evaluated, using as models various compounds characterized by a wide range of log P values. In summary, carbon-based coatings i.e., graphene, graphene oxide, MWCNTs and MWCNTs-COOH, afforded better extraction capabilities towards compounds with high log P values, with hydrophobic and π-π types of interactions as the main driving forces for adsorption. Specifically, the graphene oxide coating showed better extraction performance for polar compounds owing to its hydrophilic functional groups on the surface. Nevertheless, the overall extraction efficiencies of the carbon-based coatings for all model analytes were significantly low. Two different factors are postulated to contribute to such low recoveries: (i) poor desorption efficiencies for non-polar compounds; (ii) low dispersibility in PAN slurry, which makes carbon-based nanomaterials easily stack together, thus decreasing their surface area for adsorption. In this regard, several useful strategies can be employed to improve the extraction performance of carbon-based nanomaterials. One such strategy would be to exploit surface functionalization with appropriate organic molecules or polymers through covalent or non-covalent reactions. Functionalized graphene or MWCNTs would be more suitable for analysis of polar compounds, non-polar compounds, or both. Another way to improve extraction would be through immobilization of carbon nanomaterials onto appropriate supporting substrates, e.g., silica particles, to avoid their aggregation and increase the effective adsorption area.

In comparison to carbon-based coatings, SPE-based coatings i.e., PS-DVB, C18, HLB and PBA, showed much higher extraction efficiencies toward the tested probes. In particular, PS-DVB and HLB coatings exhibited favorable ability to extract basic, neutral, and acidic compounds with a wide range of log P values at physiological pH conditions. The C18 coating was demonstrated to be useful for extraction of non-polar compounds with very high sensitivity due to its long-chain hydrophobic alkyl groups. In the present case, the PBA coating showed lower extraction efficiencies in comparison to the other SPE-based coatings, but previous work demonstrated that the PBA sorbent can be successfully used for targeted metabolomics analysis of urinary nucleosides due to selective binding of *cis*-diol groups available on the ribose sugar group under basic conditions [33]. In sum, coating selection should take into consideration both the properties of the analytes of interest as well as those of the coating. In this regard, the current research work can provide analysts with useful information regarding coating selection for TFME applications.

## Supplementary Materials

The following are available online. The file of supplementary information is available online, and contains the following information: Section S1.1: Chemicals and Materials. Section S1.2: Stock solutions and working solutions. Section S1.3: Liquid chromatography and tandem mass spectrometry conditions. Table S1: Absolute recovery and carryover data of each analyte from PS-DVB coating by use of four desorption solvents. Solvent 1: methanol/acetonitrile/water (25/25/50, *v*/*v*/*v*), solvent 2: methanol/acetonitrile/water (40/40/25, *v*/*v*/*v*), solvent 3: methanol/acetonitrile (80/20, *v*/*v*), solvent 4: acetonitrile/water (80/20, *v*/*v*). Formic acid (0.1%, *v*/*v*) was added into all the desorption solvents. Table S2: Determination of correction factors for coating comparison. Table S3: Normalized extraction efficiency of each analyte from eight thin-film coatings at various pH values. Extractions were performed in triplicate at pH 3.0, 7.4 and 10.0, using an extraction time of 2 h. Desorption was performed for 1 h using desorption solvent 2. Table S4: Physicochemical properties and suppliers of LC analytes for coating evaluation. Table S5: The ratio of adsorbent and PAN glue for each coating. Table S6: Optimized mass spectrometry conditions for all the analytes in API 4000 mass spectrometer. Table S7: Summary of optimized LC-MS/MS parameters.
