# Supplementary material for: Systematic Evaluation of Different Coating Chemistries Used in Thin-Film Microextraction
Source: Molecules. 2020 Jul 29;25(15):3448. doi: 10.3390/molecules25153448 (PMC7435592; doi:10.3390/molecules25153448)
Supplement: Supplementary file 1 [file molecules-25-03448-s001.pdf]

Supplementary Materials

Systematic Evaluation of Different Coating Chemistries Used in Thin-Film Microextraction

Jia-wei Liu†, Khaled Murtada†, Nathaly Reyes-Garcés[1] and Janusz Pawliszyn*

Department of Chemistry, University of Waterloo, 200 University Avenue West, Waterloo, Canada, N2L 3G1; liujiawei19871995@163.com (J.L.); kmurtada@uwaterloo.ca (K.M.); nathalyreyesg@gmail.com (N.R.); janusz@uwaterloo.ca (J.P.)

[1] Current address: Restek Corporation, Bellefonte, PA, USA.

* Correspondence: janusz@uwaterloo.ca; Tel.: +1-519-888-4641 (J.P.)

† These authors contributed equally to this work.

Table of contents:

Section S1.1. Chemicals and Materials.

Section S1.2. Stock solutions and working solutions.

Section S1.3. Liquid chromatography and tandem mass spectrometry conditions.

Table S1. Absolute recovery and carryover data of each analyte from PS-DVB coating by use of four desorption solvents. Solvent 1: methanol/acetonitrile/water (25/25/50, *v*/*v*/*v*), solvent 2: methanol/acetonitrile/water (40/40/25, *v*/*v*), solvent 3: methanol/acetonitrile (80/20, *v*/*v*), solvent 4: acetonitrile/water (80/20, *v*/*v*). Formic acid (0.1%, *v*/*v*) was added into all the desorption solvents.

Table S2. Determination of correction factors for coating comparison.

Table S3. Normalized extraction efficiency of each analyte from eight thin-film coatings at various pH values. Extractions were performed in triplicate at pH 3.0, 7.4 and 10.0, using an extraction time of 2 h. Desorption was performed for 1 h using desorption solvent 2.

Table S4. Physicochemical properties and suppliers of LC analytes for coating evaluation.

Table S5. The ratio of adsorbent and PAN glue for each coating.

Table S6. Optimized mass spectrometry conditions for all the analytes in the API 4000 mass spectrometer.

Table S7. Summary of optimized LC-MS/MS parameters.

Section S1.1 – Chemicals and Materials

The selected probes comprised a diverse set of compounds covering a broad range of polarities (log P values from −2.99 to 6.98) and bearing on their structures different moieties. As can be seen in Table S1, classes of model analytes included amino acids, small peptides, nucleoside bases, steroids, alkaloids, basic drugs, fatty acids, vitamins, pesticides, carboxylic acids and PAHs. Diazepam-d5 (Cerilliant, Round Rock, TX, USA) and gemfibrozil (Sigma-Aldrich, Oakville, ON, Canada) were used as internal standards for positive and negative modes, respectively. LC-MS grade methanol and acetonitrile (Sigma-Aldrich, Oakville, ON, Canada) were employed as mobile phases and desorption solvents. LC-MS-grade ammonium formate and formic acid were obtained from Fluka (Oakville, ON, Canada). Sodium phosphate dibasic, potassium phosphate monobasic and citric acid were purchased from Sigma-Aldrich (Oakville, ON, Canada). Sodium tentraborate was obtained from Fisher Scientific (Nepean, ON, Canada), and sodium hydroxide was purchased from Caledon Laboratories Ltd. (Georgetown, ON, Canada). All the chemicals were used as received without further purification.

Graphene nanopowder (particle diameter 15 μm, thickness 6–8 nm) was obtained from SkySpring Nanomaterials, Inc. (Houston, TX, USA). Graphene oxide was home-made based on a modified Hummer's method [1]. MWCNTs (diameter 20–30 nm, length 0.5–2 μm) was purchased from Nanostructured & Amorphous Materials, Inc. (Houston, TX, USA). MWCNTs-COOH (diameter 20–40 nm, length <5 μm) was purchased from Shenzhen Nanotech. Prot. Co., Ltd. (Shenzhen, China). PS-DVB (Chromabond® Easy) particles (80 μm particle size), and phenyl boronic acid (PBA) (Agilent technology, Bond Elut PBA) modified silica particles (80 μm particle size) were obtained from VWR International (ON, Canada). Oasis HLB (polymeric reversed-phase) particles (30 μm particle size) were purchased from Waters (Oakville, ON, Canada). C18-based silica particles (5 μm particle size) were kindly supplied by Supelco (Oakville, ON, Canada). Polyacrylonitirile (PAN), N,N-dimethylformamide (DMF) and hydrochloric acid were obtained from Sigma-Aldrich (Oakville, ON, Canada).

Section S1.2 – Stock solutions and working solutions

Standard and internal standard stock solutions (1000 μg mL^{-1}) were prepared by spiking each compound either in methanol or in water, depending on each compound's solubility. A 10 μg mL^{-1} stock mixture containing all the model analytes was obtained by diluting standard stock solutions with methanol/water (50/50, *v/v*). Evaluation of the different coatings was carried out by extracting the model analytes from three buffers adjusted at different pH values: phospahate-citrate (pH 3), phosphate (pH 7.4) and borate-NaOH (pH 10). For this purpose, each buffer type was spiked with the model analytes at a concentration of 100 ng mL^{-1}, always keeping the organic solvent content below 1% (*v/v*).

Section S1.3 – Liquid chromatography and tandem mass spectrometry conditions

An Applied Biosystems API 4000 triple quadrupole mass spectrometer (equipped with TurboIonSpray source) was used in positive and negative modes and operated under multiple reaction monitoring (MRM) conditions. Separation of analytes was performed on a Shimadzu (LC-10 AD) high-pressure liquid chromatography (HPLC) coupled with a CTC PAL auto injector from Leap Technologies (CTC Analytics, NC).

Table S1. Absolute recovery and carryover data of each analyte from PS-DVB coating by use of four desorption solvents. Solvent 1: methanol/acetonitrile/water (25/25/50, *v*/*v*/*v*), solvent 2: methanol/acetonitrile/water (40/40/25, *v*/*v*), solvent 3: methanol/acetonitrile (80/20, *v*/*v*), solvent 4: acetonitrile/water (80/20, *v*/*v*). Into all the desorption solvents, 0.1% (*v*/*v*) of formic acid was added.

Analyte	Solvent 1		Solvent 2		Solvent 3		Solvent 4	
	Recovery%	Carryover%	Recovery%	Carryover%	Recovery%	Carryover%	Recovery%	Carryover%
Ala-Ala	<0.1	N.D.[a]	<0.1	N.D.	<0.1	N.D.	<0.1	N.D.
Alanine	<0.1	N.D.	<0.1	N.D.	<0.1	N.D.	<0.1	N.D.
Methionine	5.7	N.D.	0.8	N.D.	4.9	N.D.	<0.1	N.D.
Riboflavin	80.6	2.1	64.1	0.7	57.5	2.5	13.6	0.9
Phenylalanine	78.4	N.D.	63.5	0.3	56.1	0.6	14.7	8.9
Tryptophan	57.0	2.4	48.9	2.6	50.8	4.9	51.0	8.9
Adenine	46.7	0.4	48.5	0.5	46.1	1.2	50.6	0.3
Caffeine	74.3	2.3	84.2	1.0	72.6	2.2	17.1	1.0
Aspatame	72.9	4.6	64.4	6.2	52.9	9.7	66.9	8.3
Atenolol	88.7	1.9	79.1	1.5	77.8	2.3	81.5	5.5
Madelic acid	2.5	N.D.	9.2	N.D.	10.8	N.D.	4.1	N.D.
Morphine	78.5	2.1	74.3	2.0	70.8	3.2	65.0	6.4
Pindolol	87.5	2.1	93.8	1.2	83.7	1.3	76.8	3.1
Dexamethasone	84.9	19.2	78.0	4.1	70.8	3.0	64.3	1.9

3-phenylpropionic acid	30.0	N.D.	28.6	N.D.	17.7	N.D.	33.2	N.D.
Carbamazepine	94.6	13.7	98.0	4.2	100	3.1	100	3.0
Thiabendazole	90.9	11.4	93.1	11.6	78.0	18.3	86.8	15.3
Diazepam	77.4	27.7	86.6	11.8	81.3	7.9	84.0	8.6
Testosterone	65.1	25.4	79.5	8.1	72.6	5.7	74.1	5.5
Propanolol	87.8	5.4	87.5	1.9	82.0	3.1	86.9	7.8
Phenanthrene	94.0	1.6	80.0	1.0	78.9	2.4	83.3	5.1
Arachidonic acid	<0.1	N.D.	22.1	16.6	17.6	5.4	22.7	9.2

a: Not detected

Table S2. Determination of correction factors for coating comparison.

Coating	Thickness (mm)	Length (mm)	Blade thickness (mm)	Total thickness (mm)	Blade width (mm)	Total width (mm)	Total volume (mm3)	Blade volume (mm^3)	Coating volume (mm^3)	Correction factor
Graphene	0.190	20	0.494	0.684	2.132	2.266	31.00	21.06	9.94	0.77
Graphene oxide	0.139	20	0.245	0.384	1.730	1.848	8.477	14.19	5.71	1.35
MWCNTs-COOH	0.169	20	0.293	0.462	1.886	1.940	11.05	17.93	6.88	1.12
MWCNTs	0.165	20	0.261	0.426	1.659	1.981	8.66	16.34	7.68	1.00
PS-DVB	0.498	20	0.311	0.809	1.847	2.247	11.49	36.36	24.87	0.31
C18	0.206	20	0.487	0.693	2.099	2.388	20.44	33.10	12.66	0.61
HLB	0.403	20	0.506	0.909	2.158	2.622	21.84	47.67	25.83	0.30
PBA	0.872	20	0.432	1.034	2.009	2.586	17.36	53.48	36.12	0.21

Description: Since the amount of analyte extracted via SPME is dependent on the volume of the extraction phase, an objective comparison of the investigated coatings was made possible by normalizing the absolute recoveries by the coating volumes. As a matter of fact, volume variability among coatings prepared with distinct sorbents should be expected due to differences in particle sizes and in the coating application procedure itself. Therefore, extraction phase volumes were estimated by simply treating all types of coatings as cuboids and assuming that the entire volume of the immobilized phase could act as a sorbent. Volumes of two cuboids were calculated: (i) the volume of the bare blade and (ii) the volume of the coated blade. The coating volume was then determined by subtracting the bare blade volume from the total volume of the coated blade. Results corresponding to calculations of coating volumes are summarized in Table S6. Herein, the coating volume of MWCNTs was selected as reference, and the correction factors were calculated by dividing each

coating volume by the volume of the MWCNTs coating. Then, the normalized extraction efficiency of each coating for a given compound was calculated by multiplying the corresponding correction factor by the extraction efficiency of each coating for said compound.

Table S3. Normalized extraction efficiency of each analyte from eight thin-film coatings at various pH values. Extractions were performed in triplicate at pH 3.0, 7.4 and 10.0, using an extraction time of 2 h. Desorption was performed for 1 h using desorption solvent 2.

**Analyte**	**Graphene**			**Graphene oxide**			**MWCNTs-COOH**			**MWCNTs**		
	pH 3.0	pH 7.4	pH 10.0	pH 3.0	pH 7.4	pH 10.0	pH 3.0	pH 7.4	pH 10.0	pH 3.0	pH 7.4	pH 10.0
Ala-Ala	0.20%	N.D.	0.20%	0.85%	N.D	0.74%	0.25%	N.D	0.23%	0.29%	N.D	0.15%
Alanine	1.88%	N.D.	1.21%	2.72%	N.D	3.05%	2.21%	N.D	1.97%	0.94%	N.D	1.05%
Methionine	N.D.	N.D.	N.D.	N.D.	N.D	N.D	N.D	N.D	N.D	N.D	N.D	N.D
Riboflavin	0.35%	0.22%	0.07%	6.29%	6.48%	1.26%	0.31%	5.19%	0.09%	0.34%	0.13%	0.10%
Phenylalanine	0.29%	0.16%	0.25%	0.79%	0.24%	0.92%	0.05%	0.15%	0.15%	0.05%	0.14%	0.06%
Tryptophan	0.30%	0.15%	0.49%	2.19%	0.60%	1.59%	0.25%	N.D	0.26%	0.09%	N.D	0.20%
Adenine	0.58%	0.23%	0.57%	6.32%	0.88%	3.10%	0.42%	0.11%	0.66%	0.51%	0.11%	0.61%
Caffeine	0.28%	0.10%	0.22%	3.36%	1.31%	2.48%	0.17%	N.D	0.27%	0.18%	0.13%	0.16%
Aspatame	0.21%	0.13%	N.D.	1.09%	0.13%	N.D	0.11%	N.D	N.D	N.D	N.D	N.D
Atenolol	0.20%	0.34%	0.39%	2.01%	1.53%	2.14%	0.17%	0.11%	0.31%	0.10%	0.16%	0.22%
Madelic acid	N.D.	1.78%	14.0%	N.D.	1.17%	21.9%	1.99%	1.19%	23.7%	1.94%	0.90%	18.4%
Morphine	0.32%	0.19%	0.24%	2.18%	1.67%	1.51%	0.25%	0.64%	0.34%	0.21%	0.29%	0.12%
Pindolol	0.32%	0.28%	0.81%	3.23%	2.90%	5.01%	0.35%	0.10%	0.85%	0.26%	0.21%	0.62%
Dexamethasone	0.34%	0.20%	0.43%	2.09%	0.58%	1.49%	0.20%	0.15%	0.20%	0.27%	0.17%	0.24%

3-phenylpropionic acid	N.D.	N.D.	N.D.	N.D.	N.D	N.D	N.D	N.D	N.D	N.D	N.D	N.D
Carbamazepine	0.42%	0.18%	0.34%	2.30%	0.50%	1.65%	0.21%	N.D	0.28%	0.18%	0.39%	0.20%
Thiabendazole	0.68%	2.96%	3.23%	13.7%	3.97%	10.9%	0.37%	1.12%	1.61%	0.27%	1.23%	1.53%
Diazepam	1.36%	1.54%	2.95%	4.42%	1.84%	4.32%	0.79%	1.26%	2.71%	0.87%	1.31%	2.18%
Testosterone	1.81%	0.72%	1.46%	6.42%	2.23%	4.63%	0.81%	0.47%	1.10%	0.93%	0.54%	0.89%
Propranolol	0.59%	1.54%	8.81%	8.22%	7.52%	15.6%	0.37%	1.08%	8.14%	0.28%	1.12%	8.06%
Phenanthrene	0.20%	0.45%	0.21%	2.01%	1.84%	2.34%	N.D	0.14%	0.20%	N.D	0.24%	0.08%
Arachidonic acid	21.8%	3.20%	0.51%	17.4%	N.D	N.D	32.0%	1.36%	0.93%	30.6%	N.D	N.D

Continued

Analyte	PS-DVB			C18			HLB			PBA		
	pH 3.0	pH 7.4	pH 10.0	pH 3.0	pH 7.4	pH 10.0	pH 3.0	pH 7.4	pH 10.0	pH 3.0	pH 7.4	pH 10.0
Ala-Ala	0.44%	N.D	0.41%	0.26%	N.D	0.21%	0.35%	N.D	0.19%	0.22%	N.D	0.17%
Alanine	0.56%	N.D	0.50%	0.65%	N.D	0.88%	0.94%	N.D	0.97%	0.52%	N.D	0.13%
Methionine	N.D	0.26%	0.28%	N.D	N.D	N.D	N.D	N.D	N.D	N.D	N.D	0.03%
Riboflavin	22.0%	19.9%	3.16%	13.6%	28.8%	1.37%	11.4%	13.4%	0.93%	2.71%	2.97%	0.24%
Phenylalanine	6.21%	19.7%	6.47%	0.66%	0.76%	0.65%	2.68%	2.02%	4.74%	0.49%	0.47%	0.35%

Tryptophan	12.8%	15.2%	11.8%	2.29%	1.48%	1.10%	7.90%	5.85%	7.95%	0.96%	0.81%	0.62%
Adenine	10.4%	15.0%	14.1%	0.69%	0.71%	0.77%	2.85%	6.33%	4.61%	0.94%	1.65%	1.13%
Caffeine	28.5%	26.1%	28.6%	5.45%	8.61%	6.30%	21.2%	18.9%	19.8%	2.63%	2.99%	2.19%
Aspatame	23.0%	20.0%	4.83%	5.93%	7.69%	1.15%	15.6%	13.2%	2.82%	0.86%	0.98%	0.05%
Atenolol	23.6%	24.5%	22.9%	3.54%	7.59%	19.5%	10.6%	22.7%	21.1%	1.22%	4.08%	4.48%
Madelic acid	3.13%	2.84%	20.9%	0.74%	0.74%	15.1%	4.68%	3.18%	18.2%	2.40%	2.66%	16.4%
Morphine	23.3%	23.0%	25.8%	2.00%	8.01%	8.59%	11.6%	21.5%	19.3%	1.41%	4.53%	2.42%
Pindolol	25.9%	29.1%	21.3%	11.2%	25.9%	39.8%	20.9%	26.7%	24.3%	1.27%	7.27%	8.19%
Dexamethasone	28.0%	24.2%	23.1%	54.7%	50.8%	50.2%	25.8%	23.5%	20.8%	5.09%	4.39%	2.55%
3-phenylpropionic acid	31.6%	8.85%	14.6%	16.2%	N.D	2.12%	29.1%	4.21%	8.01%	3.58%	0.22%	0.60%
Carbamazepine	30.7%	30.4%	30.7%	46.1%	53.3%	46.1%	29.3%	30.1%	28.4%	8.10%	7.77%	5.57%
Thiabendazole	30.2%	28.8%	29.8%	12.4%	24.6%	21.0%	28.1%	28.2%	28.4%	3.12%	8.57%	6.24%
Diazepam	27.8%	26.8%	26.2%	47.0%	49.8%	48.5%	26.3%	25.3%	24.6%	8.92%	9.96%	8.41%
Testosterone	25.9%	24.6%	23.9%	51.4%	49.2%	48.5%	26.2%	24.1%	22.7%	9.09%	8.91%	6.62%
Propranolol	28.9%	27.1%	15.3%	42.1%	47.8%	50.5%	25.9%	26.9%	19.9%	5.53%	12.0%	10.4%
Phenanthrene	16.3%	24.8%	16.4%	3.25%	7.50%	18.6%	16.3%	23.3%	15.6%	1.07%	4.12%	4.28%
Arachidonic acid	3.21%	6.86%	7.63%	6.74%	12.8%	17.1%	3.99%	9.30%	9.12%	1.79%	1.26%	2.04%

Table S4. Physicochemical properties and suppliers of LC analytes for coating evaluation.

Compounds	Supplier	Formula	Molecular weight (g mol^{-1})	pKa1	logP1
**Alanyl-alanine (Ala-Ala)**	Sigma-Aldrich	$C_6H_{12}N_2O_3$	160.17	NA	NA
**Alanine**	Sigma-Aldrich	$C_3H_7NO_2$	89.09	2.35 (carboxyl)<br>9.69 (amino)	-2.99
**Methionine**	Sigma-Aldrich	$C_5H_{11}NO_2S$	149.21	2.28 (carboxyl)<br>9.21 (amino)	-1.87
**Riboflavin**	Fluka	$C_{17}H_{20}N_4O_6$	376.36	9.888	-1.46
**Phenylalanine**	Sigma-Aldrich	$C_9H_{11}NO_2$	165.19	1.83 (carboxyl)<br>9.13 (amino)	-1.38
**Tryptophan**	Fluka	$C_{11}H_{12}N_2O_2$	204.23	2.38 (carboxyl)<br>9.39 (amino)	-1.06
**Adenine**	Fluka	$C_5H_5N_5$	135.13	4.15 (secondary)<br>9.80 (primary)	-0.09
**Caffeine**	Sigma-Aldrich	$C_8H_{10}N_4O_2$	194.19	10.4	-0.07
**Aspartame**	Sigma-Aldrich	$C_{14}H_{18}N_2O_5$	294.31	4.11	0.07
**Atenolol**	Sigma-Aldrich	$C_{14}H_{22}N_2O_3$	266.34	9.6	0.16

**Mandelic acid**	Sigma-Aldrich	$C_8H_8O_3$	152.15	3.41	0.62
**Morphine**	Cerilliant	$C_{17}H_{19}NO_3$	285.34	8.21	0.89
**Pindolol**	Sigma-Aldrich	$C_{14}H_{20}N_2O_2$	248.32	9.25	1.75
**Dexamethasone**	Sigma-Aldrich	$C_{22}H_{29}FO_5$	392.46	NA	1.83
**3-phenylpropionic acid**	Sigma-Aldrich	$C_9H_{10}O_2$	150.18	4.66	1.84
**Carbamazepine**	Sigma-Aldrich	$C_{15}H_{12}N_2O$	236.27	NA	2.45
**Thiabendazole**	Sigma-Aldrich	$C_{10}H_7N_3S$	201.25	4.64	2.47
**Diazepam**	Cerilliant	$C_{16}H_{13}ClN_2O$	284.75	3.4	2.82
**Testosterone**	Sigma-Aldrich	$C_{19}H_{28}O_2$	288.42	NA	3.32
**Propanolol**	Sigma-Aldrich	$C_{16}H_{21}NO_2$	259.34	9.42	3.48
**Phenanthrene**	Sigma-Aldrich	$C_{14}H_{10}$	178.23	NA	4.46
**Arachidonic acid**	Cayman Chemical	$C_{20}H_{32}O_2$	304.47	4.75	6.98

[1] Syracuse Research Corporation, PhysProp Database, accessed November 2019.

Table S5. The ratio of adsorbent and PAN glue for each coating.

Coating type	Amount of adsorbent (g)	PAN glue (ca. 6.9% in w/v) volume (mL)	Ration of adsorbent/PAN (w/w)	DMF volume (mL)	Layers
Graphene-PAN	0.25	15.0	0.24	3.0	20
Graphene oxide-PAN	0.125	15.0	0.12	5.5	20
MWCNTs-COOH-PAN	0.125	15.0	0.12	5.5	12
MWCNTs-PAN	0.125	15.0	0.12	5.5	12
PS-DVB-PAN	1.0	10.0	1.45	3.0	10
C18-PAN	1.0	10.0	1.45	3.0	12
HLB-PAN	1.0	10.0	1.45	3.0	12
PBA-PAN	1.0	10.0	1.45	3.0	15

Table S6. Optimized mass spectrometry conditions for all the analytes in API 4000 mass spectrometer.

Compounds	Chemical Structure	ESI mode	Q1 mass (*m*/*z*)	Q3 mass (*m*/*z*)	DP (V)	EP (V)	CE (V)	CXP (V)
**Ala-Ala**	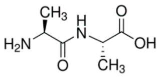	Positive	161.1	44	42	15	24	7
**Alanine**	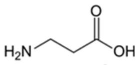	Positive	90.2	71.9	46	15	12	3
**Methionine**	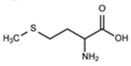	Positive	150.2	104.0	33	15	14	6
**Riboflavin**	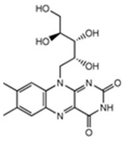	Positive	377.2	243.0	107	15	35	22
**Phenylalanine**	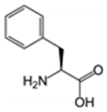	Positive	166.2	119.9	47	15	16	7
**Tryptophan**	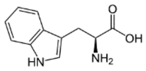	Positive	205.3	187.7	56	15	15	13
**Adenine**	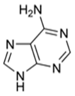	Positive	136.2	118.9	36	15	30	7
**Caffeine**	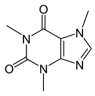	Positive	195.1	137.8	72	15	25	13
**Aspatame**	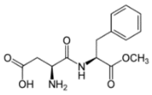	Positive	295.1	119.8	50	15	32	7
**Atenolol**	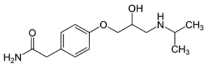	Positive	267.2	145.1	69	15	40	9
**Mandelic acid**	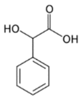	Negative	151.1	106.8	-44	-15	-13	-9
**Morphine**	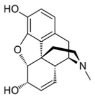	Positive	286.3	165.1	90	15	54	16
**Pindolol**	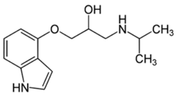	Positive	249.2	116.0	61	15	23	7

**Dexamethasone**	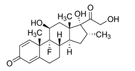	Positive	393.4	147.2	57	15	42	14
**3-phenylpropionic aicd**	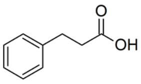	Negative	149.0	104.8	-50	-15	-15	-5
**Carbamazepine**	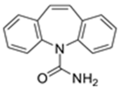	Positive	237.2	194.0	81	15	25	13
**Thiabendazole**	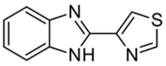	Positive	202.2	175.0	90	15	35	12
**Diazepam**	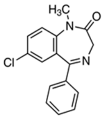	Positive	285.2	153.8	58	15	37	10
**Testosterone**	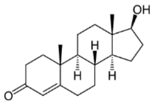	Positive	289.2	96.9	73	15	37	6
**Propanolol**	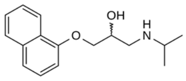	Positive	260.2	116.2	79	15	26	11
**Phenanthrene**	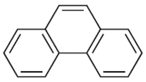	Positive	178.1	133.2	122	15	20	12
**Arachidonic acid**	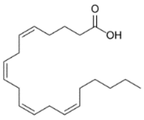	Negative	303.2	259.0	-74	-15	-16	-7

Table S7. Summary of optimized LC-MS/MS parameters.

LC-MS/MS parameter	Reverse phase LC method	
ESI mode	Positive	Negative
Analytical column	HS F5-3 Pentafluorophenyl (Supelco)	XbridgeTMC18 (Waters)
Column dimensions	2.1 µm × 15cm	
Particle size	3.0 µm	3.5µm
Mobile phase A	Water/formic acid/2 mmol L^{-1} ammonium formate (99.9/0.1, *v/v*)	Water/acetonitrile/2 mmol L^{-1} ammonium formate (90/10, *v/v*)
Mobile phase B	Acetonitrile/methanol/formic acid/2 mmol L^{-1} ammonium formate (50/49.9/0.1, *v/v*)	Acetonitrile/water/2 mmol L^{-1} ammonium formate (90/10, *v/v*)
Flow rate	0.2 mL min^{-1}	
Injection volume	20 µL	
Run time	30 min	15 min
Gradient program	0–2 min, 10% B; 2–20 min, linear gradient to 100% B; 20–25 min, hold at 100% B; 25–26 min, sharply drop to 10% B; 4 min for re-equilibration by 10% B	0–1 min, 10% B; 1–5 min, linear gradient to 100% B; 5–10 min, hold at 100% B; 10–11 min, sharply drop to 10% B, 4 min for re-equilibration by 10% B
Curtain gas	26	8
Collision gas	8	30

Ion source gas 1	25	20
Ion source gas 2	20	20
Ionspray Voltage	4500 V	−4200 V
Temperature	400 °C	

References

[1] Liu, J.W.; Zhang, Y.; Chen, X.W.; Wang, J.H. Graphene oxide-rare earth metal-organic framework composites for the selective isolation of hemoglobin. *ACS Appl. Mater. Interfaces* **2014**, *6*, 10196-10204.
